# Clinicopathological Characteristics and Overall 5-Year Survival of Colorectal Cancer: A Retrospective Study

**DOI:** 10.3390/medsci10030042

**Published:** 2022-08-09

**Authors:** Rahaf Almuhanna, Fatma Al-Thoubaity, Khadijah Almalki, Nada Algarni, Renad Hamad, Toleen Makhtoum

**Affiliations:** Department of Surgery, Faculty of Medicine, King Abdulaziz University, Jeddah 22254, Saudi Arabia

**Keywords:** colorectal cancer, 5-year survival, cancer, clinicopathological, Dukes’ stage

## Abstract

Colorectal cancer (CRC) is the third leading cause of cancer-related death. We aimed to investigate the clinicopathological characteristics and 5-year survival in CRC. This retrospective study reviewed King Abdulaziz University Hospital records from 2009 to 2019. Tumor staging was performed using Dukes’ pathological classification. Additionally, we measured the frequency of qualitative data and performed the chi-square and Mann–Whitney U-tests. Kaplan–Meier analysis was performed to calculate overall survival. Of the 574 included patients (age (mean ± standard deviation), 55.51 ± 14.28 years), 43.2% were Saudis, and most were male (58.7%). The rectum was the most common location of CRC (30.8%); 33.1% of patients presented with abdominal pain. The dominant histological variant was mucinous adenocarcinoma (95.5%). Age at diagnosis was significantly associated with Dukes’ staging; 36.3% of individuals aged <50 years had Dukes’ D stage. The 5-year survival rate was 47.9%. Better survival was noted for those of Saudi nationality, those with Dukes’ A stage, and those who were overweight (51.6%, 56.3%, and 46.8%, respectively). Significantly better survival was seen in Saudi patients due to accessible healthcare and in overweight patients due to better treatment tolerance. The outcome of CRC was not related to sex or metformin use in patients with diabetes mellitus.

## 1. Introduction

Worldwide, colon cancer is the fourth most common cancer, and rectal cancer is the eighth most common cancer. Colorectal cancer (CRC) is the most commonly diagnosed cancer and the third leading cause of death [1]. CRC is primarily known to be particularly prevalent in developed and Western countries. The Czech Republic, Australia, New Zealand, Canada, and the United States have recorded the highest incidence of CRC [2]. Treatment options are plentiful, with surgical treatment being the optimal method; other options include radiation therapy and chemotherapy [3].

Saudi Arabia, among other countries, such as Japan, Kuwait, Yemen, and South Korea, has CRC as the most diagnosed cancer in the male population [1,4] and the third most common cancer in the female population [4], with average ages of 60 and 55 years for men and women, respectively [5].

Data obtained from 1990 to 2016 show that there has been a remarkable increase in the incidence of CRC in the past 26 years in the Kingdom of Saudi Arabia (KSA) [6], as the rate is among the highest in the Gulf Cooperation Council countries [7]. Furthermore, considering the recent trend of using life expectancy, population growth, and aging to determine the potential burden of CRC, a study predicted that by 2030, the prevalence of CRC in KSA could increase four-fold in both sexes [8].

Unfortunately, the rate of death due to CRC is increasing in the KSA [9]. A factor that promotes premature mortality among Saudis is distant metastases, which approximately one-third of the patients with CRC are diagnosed with [5].

Although the mortality and incidence rates of CRC in KSA are alarmingly high [6,9], to the best of our knowledge, there are no updated studies regarding the incidence and survival of CRC at King Abdulaziz University Hospital (KAUH) in Jeddah, Saudi Arabia. Therefore, we aimed to retrospectively investigate the clinicopathological characteristics and overall 5-year survival rate of CRC at KAUH.

## 2. Materials and Methods

### 2.1. Ethics Statements

This study was approved by the research ethics committee of the Faculty of Medicine at KAUH under the supervision of the General Surgery Department (approval No.: 320-20; 8 January 2020). Informed consent was waived due to the retrospective study design.

### 2.2. Study Design and Population

This retrospective study was conducted using hospital records from 2009 to 2019 at KAUH, a tertiary center. We screened 1064 patients with CRC; after excluding patients who did not have a histopathological report or who had a colorectal tumor as a secondary lesion, the overall sample was 574 patients.

### 2.3. Data Collection

Data collected included the medical record number, date of birth, sex, nationality, age, body mass index (BMI), whether the patient was diabetic or used antidiabetic medication, presentation, whether colonoscopy was performed, tumor characteristics (location, grade, histopathology, and stage), and recurrence or metastasis, which were determined to be present or absent based on documented computerized tomographic findings or other documented medical reports. We also obtained information regarding surgery (date of the operation, type of surgery, and patient age at the time of operation). In addition, we obtained data regarding patients’ chemotherapeutic agents. To study the association between age at diagnosis and other variables, we divided the patients into age groups of <50, 50–70, and >70 years. Tumor staging was implemented according to Dukes’ classification system, a pathological staging system that classifies tumors into stages A to D depending on the extent of local involvement and regional node spread.

### 2.4. Statistical Analysis

Numbers and percentages are used to express qualitative data, and the chi-square test was performed to assess the relationship between variables. Quantitative data are expressed as mean ± standard deviation, and the Mann–Whitney U-test was performed as a nonparametric test to analyze variables that were not normally distributed to evaluate the relationships between variables. The Kaplan–Meier method was used to calculate the 5-year overall survival.

Excel 2016 (Microsoft Corp.) was used for data entry, and statistical analysis was performed using the Statistical Package for the Social Sciences (version 25; IBM Corp., Endicott, NY, USA). Statistical significance was set at a *p*-value of ≤0.05.

## 3. Results

The study included 574 patients with CRC, of whom 58.7% (337) were male and 41.3% (237) were female (Table 1). Of the total number of patients, 43.2% were Saudi and 56.8% were of different nationalities. More than half of the patients (55.2%) had an age at diagnosis ranging from 50 to 70 years with a mean of 55.51 ± 14.28 years, 29.8% had a normal BMI, and 24.7% were overweight.

Out of all patients, 181 (31.5%) had diabetes mellitus, where the most frequent treatment used was metformin 52 (9.1%), followed by a combined regime of metformin with sulfonylurea 36 (6.2%).

Histologically, 95.5% had mucinous adenocarcinoma, and more than half (67.8%) were grade 2, representing moderately differentiated tumors, followed by grade 1 (14.5%); grades 3 and 4 were found in 8.4% and 0.9% of patients, respectively. Cancer staging was performed using Dukes’ staging system; stage B was the most common stage at the time of presentation, being found in 30.1% of patients, followed by stage D (26%). Overall, 60.8% of patients had negative margins, 15.2% had lymphovascular invasion, and 13.1% had perineural invasion.

The most frequent presentation of CRC was abdominal pain (33.1%) followed by rectal bleeding (29.4%). The most common diagnostic method was colonoscopy (342 patients (59.9%)). The rectum was the most frequent location of CRC (30.8%), followed by the sigmoid colon (24.7%) and rectosigmoid junction (13.2%). At the time of surgery, the mean patient age was 56.6 ± 14.47 years. More than half of the patients showed no recurrence (52.8%). Overall, 23% patients died at a mean age of 58.41 ± 14.47 years.

A total of 400 patients received chemotherapy, 327 (57%) patients received capecitabine, 221 (38.5%) received XELOX, 60 (10%) received XELIRI, and 31 (5.4%) received FOLFOX.

The relationship between Dukes’ staging and age at diagnosis was found to be significant (*p* = 0.03), as most patients aged <50 years presented at stage D (36.3%), whereas the rest of the patients presented at stage B.

Death was associated with multiple variables, including tumor grade, Dukes’ stage, and metastasis (*p* = 0.014, *p* < 0.001, and *p* < 0.001, respectively). The percentage of death was highest in patients with Dukes’ stage D (40.3%) and grade 4 (40%), followed by those with grade 3 (35.4%). Of all the deceased patients, 34.7% had metastases. Normal-weight individuals (27.5%), followed by obese patients (25%), had a high death rate, with no significant difference (*p* = 0.34). There were no significant differences between death and sex. Additionally, no significant difference was found in the death rate between diabetic patients on metformin and those not on it (*p* = 0.78). Moreover, sex was found to have a significant relationship with Dukes’ staging (*p* = 0.05), as men accounted for the majority of stage C and D cases (27% and 26.4%, respectively).

A significant relationship was found between age at diagnosis and rectal cancer (Table 2). Rectal cancer was the most common in the age group of 50–70 years old (53.1% of patients, *p* = 0.003), with a mean age of 54.1 ± 13.1 years, whereas the mean age of patients who had tumors at other locations was 56.6 ± 13.6 years.

The overall CRC survival rate at 5 years was 47.9%. Significantly better survival was noted for those of Saudi nationality (*p* = 0.003), with a 51.6% survival rate compared with 44.7% for those with a non-Saudi nationality. The survival rate was also higher among overweight patients (46.8%, *p* = 0.001), those with Dukes’ stage A (56.3%, *p* = 0.001), those with grade 2 cancer (50.2%, *p* = 0.003), those with negative margins (63.5%, *p* = 0.001), and those without metastasis or recurrence (61.8%, *p* = 0.001) and lymphovascular or perineural invasion (58.3%, *p* = 0.001) compared with their counterparts. However, no effect of age at diagnosis or sex was observed on patients’ survival (*p* > 0.05).

## 4. Discussion

This study aimed to establish the clinicopathological characteristics of patients diagnosed with CRC at KAUH from 2009 to 2019 and to calculate the overall 5-year survival rate. According to our analysis, the majority of patients were older than 50 years of age with a mean age of 55.51 ± 14.28 years, and approximately 30% of patients diagnosed with CRC were younger than 50 years of age, corresponding to data of a national survival study in Saudi Arabia conducted from 1994 to 2004, where the mean age of patients diagnosed with CRC was 54 ± 14.7 years [9]. These findings support that patients with CRC in KSA are generally younger than those in other countries, such as England, where it has been reported that between 1996 and 2004, the mean ages at diagnosis of CRC were 68.4 years in men and 69 years in women [10]. Prevalent established CRC risk factors in the young Saudi population, such as a sedentary lifestyle, obesity, dietary habits, and smoking, can explain the high percentage of young patients with CRC [11].

In this regional study, the rectum was the most common location of CRC, followed by the sigmoid region and rectosigmoid junction, which is comparable to the findings of other regional studies [12,13]. Additionally, Dukes’ B stage represents the most frequent stage (30.1%), as reported in other studies [14]. In addition to frequency, this study points to its significant association with sex, where Dukes’ stages C and D were mainly found in men (27% and 26.4%, respectively). However, these data were not the same in a study that found an insignificant difference between the sexes, but this can be explained by the small sample size of 85 patients [15].

The present study used the Kaplan–Meier method to calculate the 5-year overall survival and analyze its association with different variables. The 5-year overall survival (47.9%) was low compared to the 69% survival rate found by the King Faisal Specialist Hospital and Research Center in Jeddah, KSA, reflecting a higher quality of care [11]. Survival was found to be better in overweight patients (46.8%); this corresponds to findings of a study in the United States dedicated to observing the correlation between BMI and long-term outcomes of patients with CRC, which indicated that it could be due to their better tolerance to cancer treatment [16]. The 5-year overall survival based on nationality was higher in Saudi patients (51.6%) than in non-Saudi patients, which could be due to the limited access of non-Saudi patients to governmental hospitals, which is the case at KAUH. Our analysis showed no significant association between sex and survival; however, conflicting data have been reported in the literature. Some studies reported that the female sex is associated with better survival, while others showed no difference [5,17,18,19]. Different variables, such as region and limited sample size, can explain this discrepancy. Several studies investigated the effect of metformin on patients with CRC overall survival. Two large studies found a significantly higher overall survival in patients on metformin [20,21]. However, other reported studies found no association between metformin use and overall survival [22,23]. In this study, no significant difference was found in the death rate between patients on metformin and patients not on this medication (*p* = 0.78), possibly due to the considerably small percent of metformin users in our sample (9%).

Regarding advanced disease presentation in KSA, in 2009–2019, 36.3% of patients who presented with Dukes’ stage D were significantly younger; this is similar to the presentation during the period of 2007–2011 in which young patients presented with an advanced stage [24]. This is in contrast to the period of 2001–2006, in which advanced stages were more frequent in older patients than in younger patients [12], supporting the change in the pattern of the disease in KSA.

### Limitations

It is worth noting that the present study did not consider patients with comorbidities that might have affected their general health, such as hypercholesterolemia or inflammatory bowel diseases or medications used in such illnesses. In addition, this study was limited to a single health center, and there was insufficient documentation of all CRC data. Thus, further studies are required to verify and validate our findings with a multicenter prospective design and to identify factors that can promote improved survival in patients with CRC.

## 5. Conclusions

Most of our patients with CRC ranged in age from 50 to 70 years, and young patients commonly presented with advanced stages of CRC. The 5-year overall survival rate was 47.9%. Saudi patients were significantly associated with better survival rates because of more accessible healthcare as well as overweight individuals due to better tolerance of cancer treatment. Additionally, this study concluded that the outcome of colorectal cancer is not related to patients’ sex or metformin use. These findings signify the need to increase public awareness, implement a national screening program, lower the threshold for suspecting CRC in younger age groups, and facilitate access to specialized medical healthcare.

## Figures and Tables

**Table 1:** Frequency of univariate sample characteristics.

Characteristic	Value	Number	Percentage (%)
Nationality	Saudi	248	43.2
	Non-Saudi	326	56.8
Sex	Female	237	41.3
	Male	337	58.7
Body mass index category	Underweight	16	2.8
	Normal	171	29.8
	Overweight	142	24.7
	Obese	124	21.6
	Insufficient data	121	21.1
Diabetics	Yes	181	31.5
	No	393	39.3
Antidiabetic agents	Insulin	24	4.2
	Metformin	52	9.1
	Sulfonylurea	12	2
	Metformin with sulfonylurea	36	6.3
	Metformin, sulfonylurea, and DPP-4 inhibitor	3	0.5
	Metformin and SGLT-2	1	0.2
	Metformin and DPP-4 inhibitor	1	0.2
	DPP-4 inhibitor	1	0.2
	SGLT-2	1	0.2
	Not on medications	9	1.6
	Insufficient data	44	7.7
	Not applicable	390	67.9
Age at diagnosis	<50 years	171	29.8
	50–70 years	317	55.2
	>70 years	82	14.3
	Insufficient data	4	0.7
Margin	Positive	26	4.5
	Negative	348	60.6
	Not applicable	153	26.7
	Not found	47	8.2
Lymphovascular invasion	Yes	87	15.2
	No	281	49
	Not applicable	151	26.3
	Not found	55	9.6
Perineural invasion	Yes	75	13.1
	No	268	46.7
	Not applicable	153	26.7
	Not found	78	13.6
Dukes’ stage	A	15	2.6
	B	173	30.1
	C	143	24.9
	D	149	26
	Insufficient data	94	16.4
Grade of cancer	Grade 1	83	14.5
	Grade 2	389	67.8
	Grade 3	48	8.3
	Grade 4	5	0.9
	Insufficient data	49	8.5
Histological finding	Mucinous adenocarcinoma	548	95.5
	Medullary carcinoma	9	1.6
	Hodgkin-like	1	0.2
	GIST	4	0.7
	Large-cell neuroendocrine carcinoma	3	0.5
	Signet ring cell carcinoma	2	0.3
	Squamous cell carcinoma	1	0.2
	Undifferentiated carcinoma	1	0.2
	Insufficient data	6	1.1
Did the patient have surgery?	Yes	419	73
	No	155	27
Location	Cecum	45	7.8
	Right colon	50	8.7
	Hepatic flexure	13	2.3
	Transverse colon	26	4.5
	Splenic flexure	11	1.9
	Left colon	43	7.5
	Sigmoid colon	42	24.7
	Rectum	177	30.8
	Rectosigmoid junction	76	13.2
	Anal canal	29	5.1
	Unspecified	17	3
Chemotherapeutic and biological agents	Oxaliplatin	1	0.2
	Capecitabine	327	57
	Fluorouracil	4	0.7
	Bevacizumab	53	9.2
	XELOX	221	38.5
	XELIRI	60	10.5
	FOLFOX	31	5.4
	FOLFIRI	7	1.2
	Trifluridine and tipiracil	2	0.3
	Panitumumab	3	0.5
	Not applicable	174	30.3
Metastasis	Yes	244	42.5
	No	330	57.5
Recurrence	Yes	271	47.2
	No	303	52.8

GIST, gastrointestinal stromal tumor. DPP-4 inhibitors, dipeptidyl peptidase-4 inhibitor. SGLT-2 inhibitors, sodium -glucose-cotransporter-2 inhibitors. XELOX, capecitabine plus oxaliplatin. XELIRI, irinotecan plus capecitabine. FOLFOX, folinic acid plus 5-fluorouracil plus oxaliplatin. FOLFIRI, leucovorin calcium plus 5-fluorouracil plus irinotecan.

**Table 2:** Relationship between patients’ outcome and their characteristics.

Variable	Deceased		χ^2^	*p*-Value
	Yes, Number (%)	No, Number (%)		
Nationality				
Non-Saudi	90 (27.6)	236 (72.4)	9.05	0.003
Saudi	42 (16.9)	206 (83.1)		
Sex				
Female	57 (24.1)	180 (75.9)	0.25	0.615
Male	75 (22.3)	262 (77.7)		
Body mass index category				
Underweight	3 (18.8)	13 (81.3)	4.52	0.34
Normal	47 (27.5)	124 (72.5)		
Overweight	26 (18.3)	116 (81.7)		
Obese	31 (25)	93 (75)		
Not applicable	25 (20.7)	96 (79.3)		
Age at diagnosis				
<50 years	67 (39.2)	104 (60.8)	38.48	<0.001
50–70 years	47 (14.8)	270 (85.2)		
>70 years	18 (22)	64 (78)		
Insufficient data	0 (0.0)	4 (100)		
Dukes’ stage				
A	0 (0.0)	15 (100)	44	<0.001
B	27 (15.6)	146 (84.4)		
C	21 (14.7)	122 (85.3)		
D	60 (40.3)	89 (59.7)		
Insufficient data	24 (25.5)	70 (74.5)		
Grade of cancer				
Grade 1	17 (20.5)	66 (79.5)	12.42	0.014
Grade 2	78 (20.1)	311 (79.7)		
Grade 3	17 (35.4)	31 (64.6)		
Grade 4	2 (40)	3 (60)		
Insufficient data	18 (36.7)	31 (63.3)		
Metastasis				
Yes	89 (36.5)	155 (63.5)	43.54	<0.001
No	43 (13)	287 (87)		
Recurrence				
Yes	94 (34.7)	177 (65.3)	39.61	<0.001
No	38 (12.5)	265 (87.5)		
Margins				
Positive	9 (34.6)	17 (65.4)	17.09	0.001
Negative	61 (17.5)	287 (82.5)		
Not applicable	51 (33.3)	102 (66.7)		
Insufficient data	11 (23.4)	36 (76.6)		
Lymphovascular invasion				
Yes	21 (24.1)	66 (75.9)	15.21	0.002
No	47 (16.7)	234 (83.3)		
Not applicable	50 (33.1)	101 (66.9)		
Insufficient data	14 (25.5)	41 (74.5)		
Perineural invasion				
Yes	15 (20)	60 (80)	16.95	0.001
No	45 (16.8)	223 (83.2)		
Not applicable	52 (34)	101 (66)		
Insufficient data	20 (25.6)	58 (74.7)		

## Data Availability

The data presented in this study are included in the tables. Additional data are available on request from the corresponding author. The data are not publicly available due to patient privacy.
